# In-hospital and 1-year outcomes of patients without modifiable risk factors presenting with acute coronary syndrome undergoing PCI: a Sex-stratified analysis

**DOI:** 10.3389/fcvm.2023.1235667

**Published:** 2023-12-20

**Authors:** Ali Sheikhy, Aida Fallahzadeh, Mana Jameie, Afsaneh Aein, Farzad Masoudkabir, Milad Maghsoudi, Masih Tajdini, Mojtaba Salarifar, Yaser Jenab, Hamidreza Pourhosseini, Mehdi Mehrani, Mohammad Alidoosti, Ali Vasheghani-Farahani, Kaveh Hosseini

**Affiliations:** ^1^ Tehran Heart Center, Cardiovascular Diseases Research Institute, Tehran University of Medical Sciences, Tehran, Iran; ^2^ Cardiac Primary Prevention Research Center, Cardiovascular Diseases Research Institute, Tehran University of Medical Sciences, Tehran, Iran; ^3^ Faculty of Medicine, Isfahan University of Medical Sciences, Isfahan, Iran

**Keywords:** acute coronary syndrome, percutaneous coronary intervention, heart disease risk factors, mortality, sex difference, health status disparities, SMuRF-less

## Abstract

**Aim:**

A considerable proportion of patients admitted with acute coronary syndrome (ACS) have no standard modifiable cardiovascular risk factors (SMuRFs: hypertension, diabetes mellitus, dyslipidemia, and cigarette smoking). The outcomes of this population following percutaneous coronary intervention (PCI) are debated. Further, sex differences within this population have yet to be established.

**Methods:**

This retrospective cohort study included 7,847 patients with ACS who underwent PCI. The study outcomes were in-hospital mortality, all-cause mortality, and major adverse cardio-cerebrovascular events (MACCE). The association between the absence of SMuRFs (SMuRF-less status) and outcomes among all the patients and each sex was assessed using logistic and Cox proportional hazard regressions.

**Results:**

Approximately 11% of the study population had none of the SMuRFs. During 12.13 [11.99–12.36] months of follow-up, in-hospital mortality (adjusted-odds ratio (OR):1.51, 95%confidence interval (CI): 0.91–2.65, *P*:0.108), all-cause mortality [adjusted-hazard ratio (HR): 1.01, 95%CI: 0.88–1.46, *P*: 0.731], and MACCE (adjusted-HR: 0.93, 95%CI:0.81–1.12, *P*: 0.412) did not differ between patients with and without SMuRFs. Sex-stratified analyses recapitulated similar outcomes between SMuRF+ and SMuRF-less men. In contrast, SMuRF-less women had significantly higher in-hospital (adjusted-OR: 3.28, 95%CI: 1.92–6.21, *P* < 0.001) and all-cause mortality (adjusted-HR:1.41, 95%CI: 1.02–3.21, *P*: 0.008) than SMuRF+ women.

**Conclusions:**

Almost one in 10 patients with ACS who underwent PCI had no SMuRFs. The absence of SMuRFs did not confer any benefit in terms of in-hospital mortality, one-year mortality, and MACCE. Even worse, SMuRF-less women paradoxically had an excessive risk of in-hospital and one-year mortality.

## Highlights

• Approximately 11% of patients with ACS undergoing non-elective PCI have none of the traditional cardiovascular risk factors.
• The risk of post-PCI in-hospital and one-year outcomes did not differ between SMuRF+ and SMuRF-less patients with ACS, indicating that the absence of traditional risk factors did not confer any additional benefit with respect to adverse outcomes following PCI.
• SMuRF-less women had higher in-hospital and all-cause mortality than SMuRF+ women, while this finding was not applicable to SMuRF-less men.

## 1. Introduction

The standard modifiable cardiovascular risk factors (SMuRFs: hypertension, diabetes mellitus, dyslipidemia, and cigarette smoking) play a role in the pathogenesis of coronary heart disease (CHD) (1). Although strategies targeted at primary and secondary prevention against these factors have contributed to a reduced risk of adverse outcomes in patients with acute coronary syndrome (ACS) (2), CHD has remained the leading cause of death in almost all regions of the world (3). The proportion of patients presenting with ACS and without SMuRFs (SMuRF-less) is increasing (4); thus, paying attention to this challenging group of patients may improve the global burden of CHD. While some large registry-based studies have evaluated the trend and outcomes of SMuRF-less patients compared with those with at least one SMuRF, the results are inconsistent (4–6). Moreover, data on SMuRF-less patients undergoing percutaneous coronary intervention (PCI) are scarce. Besides the presence or absence of conventional CHD risk factors, sex differences constitute a significant issue in determining the prognosis of patients with ACS. Despite the advances in ACS management, evident disparity exists in clinical outcomes between men and women. Many studies have assessed the impact of sex on morbidities and mortality after PCI (7), but only a few have addressed the potential role of sex in modifying post-PCI outcomes according to the presence of SMuRFs (5).

In light of this information, the present study aimed to evaluate the association between SMuRF-less status and post-PCI outcomes among patients with ACS. Further, we sought to determine possible sex differences in the post-PCI outcomes related to the SMuRF-less status.

## 2. Material and methods

### 2.1. Study design and population

The present cohort study included 8,126 patients admitted with ACS to Tehran Heart Center (THC) (8) who underwent PCI (April 2015 through December 2019). ACS comprised ST-elevation myocardial infarction (STEMI), non–ST-elevation myocardial infarction (NSTEMI), and unstable angina. Identification of patients was based on ICD-10 diagnosis codes and discharge chart reviews. Patients with inadequate data were excluded, and 7,847 patients remained for the final analysis. The patients were stratified into two groups according to their baseline risk factors status (with (SMuRF+) and without SMuRFs (SMuRF-less)). Data were retrieved from the THC database, encompassing the baseline characteristics, procedural details, and clinical outcomes during follow-ups.

### 2.2. SMuRF definition

SMuRFs were defined as dyslipidemia, hypertension, diabetes mellitus, and current cigarette smoking (5). SMuRF+ patients were those with at least one of the abovementioned risk factors. Dyslipidemia was defined as the presence of a minimum total cholesterol level of 240 mg/dl, a minimum triglyceride level of 200 mg/dl, a high-density lipoprotein cholesterol level of less than 40 mg/dl in men and less than 50 mg/dl in women, a minimum low-density lipoprotein cholesterol level of 160 mg/dl, or a history of prescribed lipid-lowering medications based on the National Cholesterol Education Program (NCEP) Adult Treatment Plan (ATP) III (9, 10). A minimum systolic blood pressure of 140 mm Hg, a minimum diastolic blood pressure of 90 mm Hg, or a history of receiving antihypertensive therapy was described as hypertension (11). Diabetes mellitus was diagnosed based on the recommendations of the American Diabetes Association if the patient had a previous history of diabetes, a minimum fasting plasma glucose level of 126 mg/dl, or a minimum 2-hour postload glucose level of 200 mg/dl (12). Current smokers were considered individuals who self-reported smoking more than 100 cigarettes in their lifetimes and who currently smoked cigarettes (within the past month).

### 2.3. ACS management and procedural technique

When diagnosed with ACS, patients were treated according to respective guidelines at the time (13, 14). PCI procedures were performed based on the standard technique. Guideline-based antiplatelet and anticoagulant therapy was applied (15). Accordingly, patients received a loading dose of a P2Y12 receptor inhibitor (300–600 mg clopidogrel, or 60 mg prasugrel, or 180 mg ticagrelor) in addition to aspirin (81–325 mg according to patient previous medical history) before the procedure and intravenous unfractionated heparin (70–100 IU/kg) during PCI. Additionally, aspirin (81 mg/day) was maintained indefinitely and P2Y12 inhibitors for at least one year (clopidogrel (75 mg, daily), or prasugrel (10 mg, daily), or ticagrelor (90 mg, twice daily).

### 2.4. Study Endpoints and Variable Definition

The study endpoints were the occurrence of in-hospital mortality, one-year all-cause mortality, and one-year major adverse cardio-cerebrovascular events (MACCE). In-hospital mortality was defined as mortality within 30 postoperative days. MACCE comprised a composite of death, nonfatal ACS, nonfatal cerebrovascular accident/transient ischemic attack, and repeated coronary revascularization via PCI/coronary artery bypass graft surgery. The definition of other variables adhered to the Catheterization and Percutaneous Coronary Intervention (CathPCI) Registry (16) and was in line with our previous studies on this population (17).

### 2.5. Ethical considerations

The current study was performed in accordance with the Declaration of Helsinki 2013. The study protocol was approved by the Ethics Committee of THC (ethics approval number: IR-THC-13799 on 26 November 2020). The Ethics Committee waived the need for Informed consent.

### 2.6. Statistical analysis

Means with standard deviations (SDs) and medians with 25th and 75th percentiles [interquartile range (IQR) boundaries] were reported for continuous variables with normal and skewed distributions, respectively. The normality of the variables was assessed using histogram charts in addition to central tendency and dispersion measures. The continuous variables were compared using the Student *t* test if they had normal distributions and the Mann–Whitney *U* test if they had skewed distributions. Categorical variables were expressed as frequencies and percentages and were compared using the Chi-squared test. Odds ratios (ORs) and Hazard ratios (HRs) with 95% confidence intervals (CIs) were calculated using binary logistic and Cox proportional hazard models. A separate landmark analysis with the landmark (cutoff) set at 30 days after the index procedure was performed as well. All the models were adjusted for age, sex, body mass index (BMI), chronic obstructive pulmonary disease, cerebrovascular accident, previous myocardial infarction, estimated glomerular filtration rate, number of diseased vessels, ejection fraction, and left main disease. Binary logistic regression was also used to assess the association between SMuRF-less status and in-hospital mortality. The analyses were performed for the total study cohort and each sex.

The statistical analyses were conducted applying R version 4.0.3 using several packages, including “survival" (18), “survminer" (19), and “ggplot2” (20). A 2-sided *P* value of less than 0.05 was considered to indicate statistical significance.

## 3. Results

### 3.1. Study population

A total of 7,847 patients [median follow-up = 12.13 (11.99–12.36) months] comprised the final study cohort: among whom 853 patients (10.9%) belonged to the SMuRF-less.

### 3.2. Baseline characteristics

The study population's baseline and procedural characteristics are presented in Table 1. In brief, SMuRf-less women tended to be younger (63.43 ± 12.62 vs. 66.51 ± 10.06), with a lower prevalence of BMI > 30 (33.6% vs. 43.9%), while they had a higher prevalence of chronic obstructive pulmonary disease (6.3% vs. 3.0%), left artery disease (63.0% vs. 49.6%), and single-vessel disease (52.8% vs. 38.5%) than SMuRF+ women. SMuRF-less men were more likely to be older (62.88 ± 12.70 vs. 60.70 ± 11.14), have higher rates of left artery disease (53.3% vs. 46.7%), and single-vessel disease (45.8% vs. 36.5%). In contrast, they were less likely to have BMI > 30 (18.9% vs. 25.4%), a positive family history of premature coronary artery disease (12.9% vs. 17.0%), a history of cerebrovascular diseases (1.4% vs. 3.2%), and exhibit a distribution of three-vessel disease (20.0% vs. 27.4%) than their SMuRF+ counterparts.

**Table 1:** Baseline and procedural characteristics.

		All patients (7,847)			Female (1,876, 23.9%)			Male (5,971, 76.1%)		
		SMuRF-less (853, 10.9%)	SMuRF+ (6,994, 89.1%)	*P*-value	SMuRF-less (127, 6.8%)	SMuRF+ (1,749, 93.2%)	*P*-value	SMuRF-less (726, 12.2%)	SMuRF+ (5,245, 87.8%)	*P*-value
Age, years		63.10 ± 8.91	62.53 ± 10.91	0.213	63.43 ± 12.62	66.51 ± 10.06	0.008	62.88 ± 12.70	60.70 ± 11.14	<0.001
BMI > 30 Kg/m^2^		178 (21.1%)	2,085 (30.0%)	<0.001	42 (33.6%)	762 (43.9%)	0.024	136 (18.9%)	1,323 (25.4%)	<0.001
Dyslipidemia		NA	4,493 (64.43%)	–	–	1,305 (74.6%)	NA	–	3,188 (60.8%)	NA
Hypertension		NA	3,911 (55.9%)	–	–	1,392 (79.6%)	NA	–	2,519 (48.0%)	NA
Diabetes mellitus		NA	2,952 (42.2%)	–	–	993 (56.8%)	NA	–	1,959 (37.3%)	NA
Current cigarette smoking		NA	2,549 (36.4%)	–	–	111 (6.3%)	NA	–	2,438 (46.5%)	NA
Positive family history		115 (13.5%)	1,238 (17.7%)	0.002	21 (16.5%)	348 (19.9%)	0.357	94 (12.9%)	890 (17.0%)	0.006
Opium use	Current	40 (4.7%)	836 (12.0%)	<0.001	0 (0.0%)	35 (2.0%)	0.245	40 (5.5%)	801 (15.3%)	<0.001
	Former	28 (3.3%)	365 (5.2%)		0 (0.0%)	3 (0.2%)		28 (3.9%)	362 (6.9%)	
Heart failure		28 (3.3%)	190 (2.7%)	0.342	5 (3.9%)	47 (2.7%)	0.407	23 (3.2%)	143 (2.7%)	0.498
Cerebrovascular disease		14 (1.6%)	254 (3.6%)	0.013	4 (3.1%)	87 (5.0%)	0.355	10 (1.4%)	167 (3.2%)	0.007
COPD		21 (2.5%)	145 (2.1%)	0.449	8 (6.3%)	53 (3.0%)	0.045	13 (1.8%)	92 (1.8%)	0.944
eGFR, ml/min/1.73m^2^				0.412			0.093			0.097
<60		131 (15.8%)	1,142 (16.5%)		25 (20.3%)	451 (26.0%)		106 (15.0%)	691 (13.3%)	
60–90		284 (34.3%)	2,311 (33.3%)		42 (34.1%)	656 (37.9%)		242 (34.3%)	1,655 (31.8%)	
>90		413 (49.9%)	3,483 (50.2%)		56 (45.5%)	625 (36.1%)		357 (50.6%)	2,858 (54.9%)	
LM lesion		46 (5.4%)	468 (6.7%)	0.148	5 (3.9%)	103 (5.9%)	0.362	41 (5.6%)	365 (7.0%)	0.188
Treated vessel	LAD	467 (56.4%)	3,316 (47.4%)	<0.001	80 (63.0%)	867 (49.6%)	0.062	387 (53.3%)	2,449 (46.7%)	0.021
	RCA	233 (27.3%)	1,911 (27.3%)		26 (20.5%)	453 (25.9%)		207 (28.5%)	1,458 (27.8%)	
	LCX	118 (13.8%)	1,362 (19.5%)		19 (15.0%)	341 (19.5%)		99 (13.6%)	1,021 (19.5%)	
	SVG	23 (2.7%)	306 (4.4%)		1 (0.8%)	61 (3.5%)		22 (3.0%)	245 (4.7%)	
	Ramus	9 (1.1%)	59 (0.8%)		1 (0.8%)	13 (0.7%)		8 (1.1%)	46 (0.9%)	
	LM	3 (0.4%)	40 (0.6%)		0	14 (0.8%)		3 (0.4%)	26 (0.5%)	
Preprocedural TIMI flow	0	301 (35.3%)	2,285 (32.7%)	0.496	42 (33.1%)	498 (28.5%)	0.268	259 (35.7%)	1,787 (34.1%)	0.818
	1	37 (4.3%)	316 (4.5%)		4 (3.1%)	70 (4.0%)		33 (4.5%)	246 (4.7%)	
	2	133 (15.6%)	984 (14.1%)		27 (21.3%)	231 (13.2%)		106 (14.6%)	753 (14.4%)	
	3	382 (44.8%)	3,409 (48.7%)		54 (42.5%)	950 (54.3%)		328 (45.2%)	2,459 (46.9%)	
Door to needle, min		55 [51–61]	54 [52–55]	0.938	51 [45, 71]	57 [55, 60]	0.307	55 [50, 60]	53 [51, 55]	0.649
ACC/AHA	A	1 (0.1%)	12 (0.2%)	0.541	0	2 (0.1%)	0.470	1 (0.1%)	10 (0.2%)	0.724
	B1	138 (16.2%)	1,217 (17.4%)		26 (20.5%)	343 (19.6%)		112 (15.5%)	874 (16.7%)	
	B2	127 (14.9%)	927 (13.3%)		23 (18.1%)	236 (13.5%)		104 (14.3%)	691 (13.2%)	
	C	587 (68.8%)	4,838 (69.2%)		78 (61.4%)	1,168 (66.8%)		509 (70.1%)	3,670 (70.0%)	
Number of diseased vessels	2VD	289 (33.9%)	2,466 (35.3%)	<0.001	41 (32.3%)	572 (32.7%)	0.003	248 (34.2%)	1,894 (36.1%)	<0.001
	3VD	164 (19.2%)	1,939 (27.7%)		19 (15.0%)	503 (28.8%)		145 (20.0%)	1,436 (27.4%)	
	SVD	400 (46.9%)	2,589 (37.0%)		67 (52.8%)	674 (38.5%)		333 (45.8%)	1,916 (36.5%)	
EF < 40%		277 (32.5%)	2,086 (29.8%)	0.114	35 (27.6%)	518 (29.6%)	0.687	242 (33.3%)	1,568 (29.9%)	0.064

BMI, body mass index; COPD, chronic obstructive pulmonary disease; eGFR, estimated glomerular filtration rate; LM, left stenosis; LAD, left anterior descending artery; RCA, right coronary artery; LCX, left circumflex artery; SVG, saphenous vein grafts; TIMI, thrombolysis in myocardial infarction; 2VD, two-vessel disease; 3VD, three-vessel disease; SVD, single-vessel disease; EF, ejection fraction.

### 3.3. In-hospital mortality

**Figure 1:**
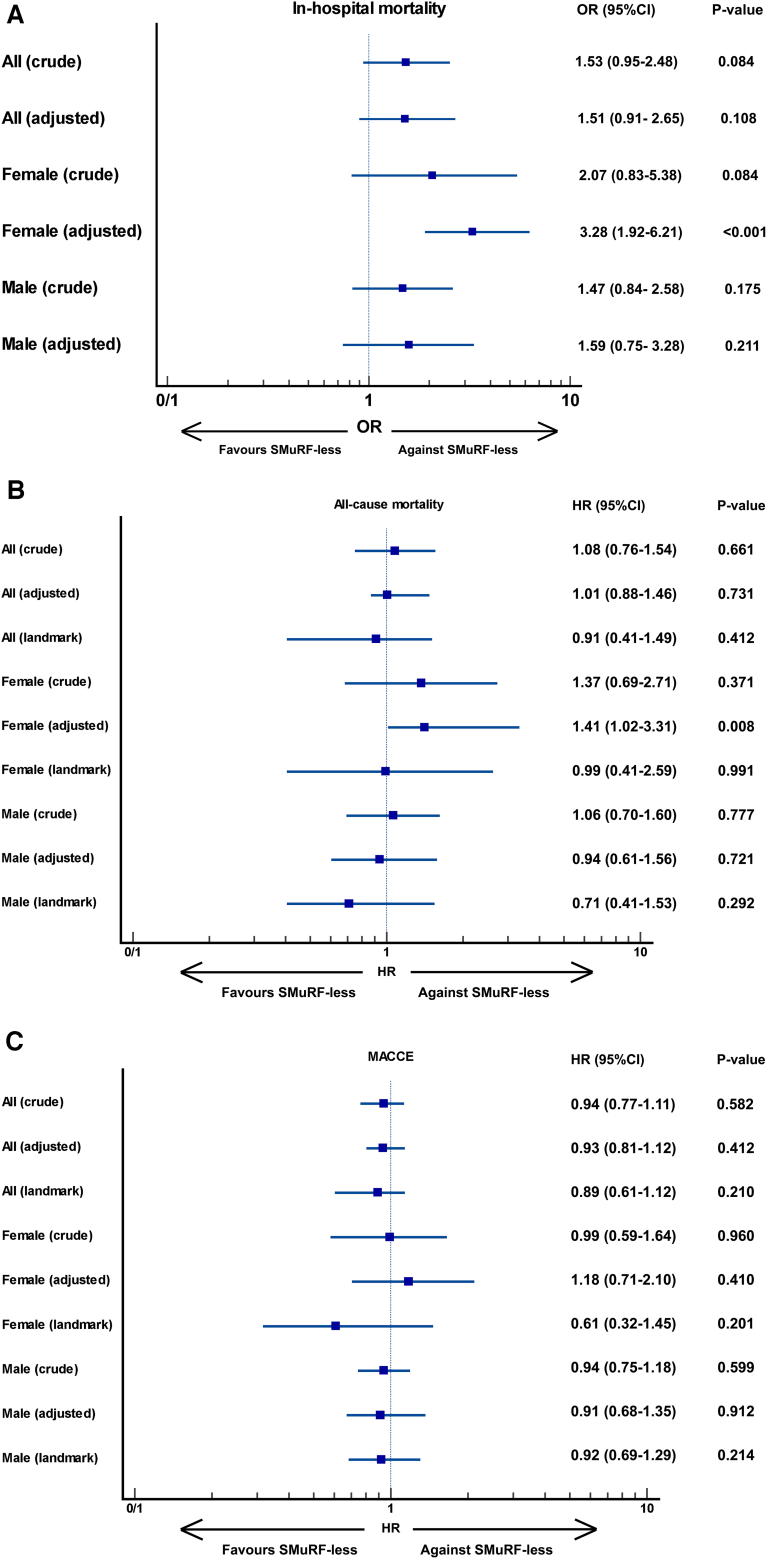
The association between the sMuRF-less status and (**A**) in-hospital mortality, (**B**) all-cause mortality, and (**C**) MACCE, overall and by sex. MACCE, Major adverse cardio-cerebrovascular events.

A total of 125 in-hospital mortalities occurred: 20 (2.3%) in the SMuRF-less and 105 (1.5%) in the SMuRF+ group. The highest in-hospital mortality rate was related to SMuRF-less women (3.9%), followed by SMuRF-less men (2.1%), SMuRF+ women (1.9%), and SMuRF+ men (1.4%) (Table 2). At the unadjusted level, the in-hospital mortality rate of SMuRF-less patients did not differ from that of the SMuRF+ group, either overall (OR: 1.53, 95%CI: 0.95–2.48, *P*: 0.084) or sex-stratified (women: OR: 2.07, 95%CI:0.83–5.38, *P*: 0.084; men: OR: 1.47, 95%CI: 0.84–2.58, *P*: 0.175) (Figure 1). After adjustments, SMuRF-less women had a significantly enhanced risk of in-hospital mortality (adjusted-OR: 3.28, 95%CI: 1.92–6.21, *P* < 0.001), while this did not apply to SMuRF-less men (adjusted-OR: 1.29, 95%CI: 0.75–3.28, *P*: 0.211).

**Table 2:** The number of events according to the presence of SMuRF, overall and by sex.

		All patients		Female		Male	
		SMuRF-less (*n* = 853)	SMuRF+ (*n* = 6,994)	SMuRF-less (*n* = 127)	SMuRF+ (*n* = 1,749)	SMuRF-less (*n* = 726)	SMuRF+ (*n* = 5,245)
All-cause mortality	Total	35 (4.1%)	273 (3.9%)	9 (7.1%)	90 (5.1%)	26 (3.6%)	183 (3.5%)
	Landmark^a^	13 (1.5%)	150 (2.3%)	4 (3.3%)	50 (3.0%)	9 (1.3%)	100 (2.0%)
MACCE (first events)	Total	99 (11.6%)	898 (12.8%)	16 (12.6%)	226 (12.9%)	83 (11.4%)	672 (12.8%)
	Landmark^a^	42 (4.9%)	488 (7.0%)	4 (3.5%)	122 (7.5%)	38 (5.7%)	366 (7.5%)
Death	Total	34 (4.0%)	264 (3.8%)	8 (6.3%)	90 (5.1%)	26 (3.6%)	174 (3.3%)
ACS	Total	16 (1.9%)	241 (3.4%)	1 (0.8%)	42 (2.4%)	15 (2.1%)	199 (3.8%)
CVA/TIA	Total	0	5 (0.1%)	0	1 (0.1%)	0	4 (0.1%)
Repeated revascularization	Total	49 (5.7%)	389 (5.6%)	7 (5.5%)	94 (5.4%)	42 (5.8%)	295 (5.6%)
In-hospital mortality	NA (total)	20 (2.3%)	105 (1.5%)	5 (3.9%)	34 (1.9%)	15 (2.1%)	71 (1.4%)

MACCE, major adverse cardio-cerebrovascular events; ACS, acute coronary syndrome; CVA, cerebrovascular accidents; TIA, transient ischemic attack.

^a^ Patient who were deceased within 30 post-operative days were excluded from the denominator in the landmark analyses.

### 3.4. One-year all-cause mortality

**Figure 2:**
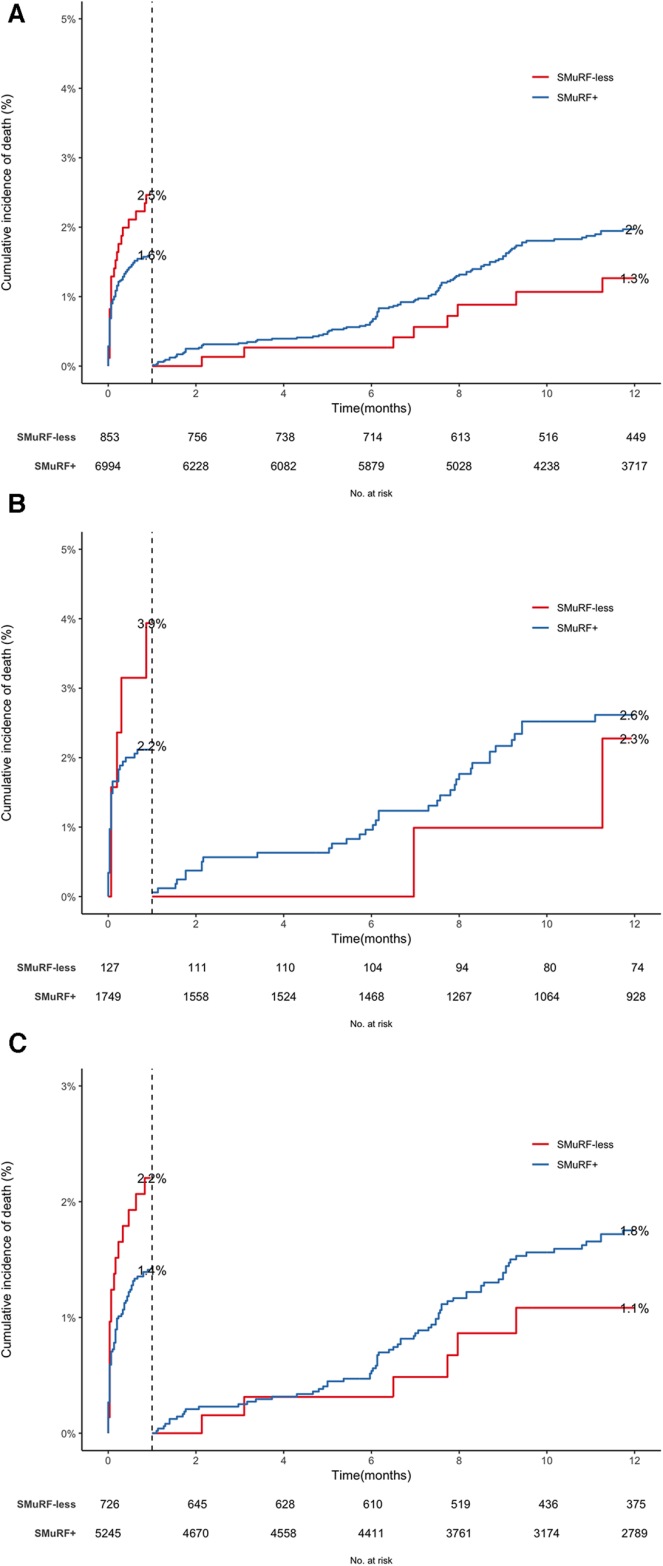
Cumulative hazard function curves of all-cause mortality for (**A**) all the patients, (**B**) women, and (**C**) men.

In the study population, 308 all-cause mortalities occurred [35 (4.1%) and 273 (3.9%) in the SMuRF-less and SMuRF+ groups, respectively]. SMuRF-less women experienced the highest all-cause mortality, followed by SMuRF+ women, SMuRF-less men, and SMuRF+ men (Table 2). Figure 2 depicts the survival curves of all-cause mortality. Among the total population and each sex, early mortality was higher among SMuRF-less patients (especially evident in women). Nevertheless, the pattern was reversed after landmark analyses (cutoff: 30 days), indicating that a considerable mortality rate among SMuRF-less patients were related to in-hospital events. According to Cox proportional hazard models (Figure 1), one-year mortality did not differ between SMuRF+ patients at the unadjusted level, either for the total population or each sex. After adjustments, mortality risk was significantly higher by 41% among SMuRF-less women than among SMuRF+ women (adjusted-HR: 1.41, 95%CI: 1.02–3.31, *P*: 0.008). This observation did not apply to male patients (adjusted-HR: 0.94, 95%CI: 0.61–1.56, *P*: 0.721) or the overall population (adjusted-HR: 1.01, 95%CI: 0.88–1.46, *P*: 0.731). Following landmark analyses, the significant association among female patients disappeared, implying that the significance of all-cause mortality stemmed from higher in-hospital mortality among SMuRF-less women (as stated above).

### 3.5. One-year MACCE

**Figure 3:**
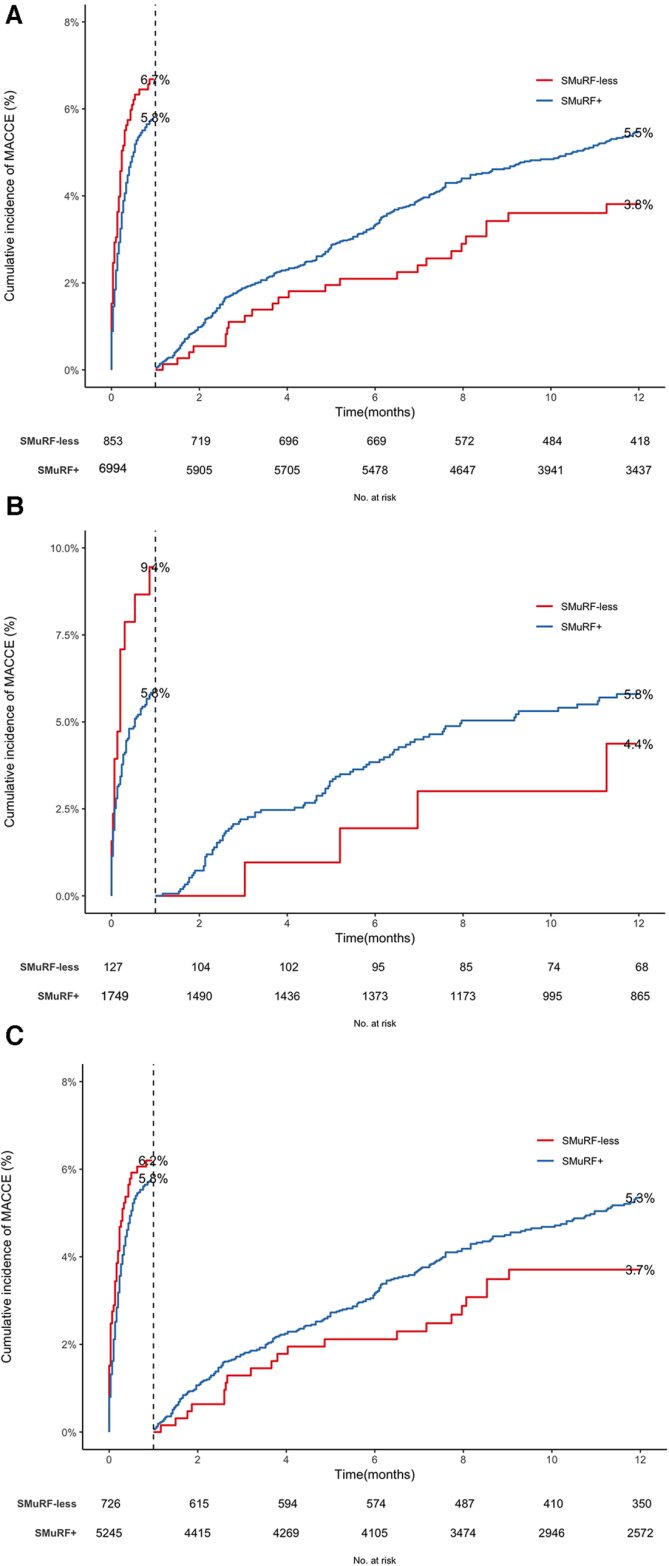
Cumulative hazard function curves of MACCE for (**A**) all the patients, (**B**) women, and (**C**) men. MACCE, Major adverse cardio-cerebrovascular events.

Totally, 997 MACCE events occurred: 99 (11.6%) in SMuRF-less and 898 (12.8%) in SMuRF+ patients, with SMuRF+ women having the most and SMuRF-less men the fewest events (Table 2). According to survival curves and in line with mortality curves (Figure 3), early MACCE events afflicted SMuRF-less patients more frequently. However, following landmark analyses, the SMuRF+ group experienced more events. The unadjusted Cox proportional hazard model recapitulated these findings, reporting comparable MACCE risks between SMuRF+ and SMuRF-less patients, overall and by sex (overall: HR: 0.94, 95%CI: 0.77–1.11, *P*: 0.582; female: HR: 0.99, 95%CI: 0.59–1.64, *P*:0.960; male: HR: 0.94, 95%CI: 0.75–1.18, *P*: 0.599). Likewise, the adjusted MACCE risk did not differ between the two groups, whether for the total population (adjusted-HR: 0.93, 95%CI: 0.81–1.12, *P*: 0.412), women (adjusted-HR: 1.18, 95%CI: 0.71–2.10, *P*: 0.410), or men (adjusted-HR: 0.91, 95%CI: 0.68–1.35, *P*:0.912). Albeit nonsignificant, the hazards were considerably attenuated, even reversed for women, after landmark analyses, suggesting that a considerable proportion of MACCEs were related to the first 30-day death.

## 4. Discussion

**Figure 4:**
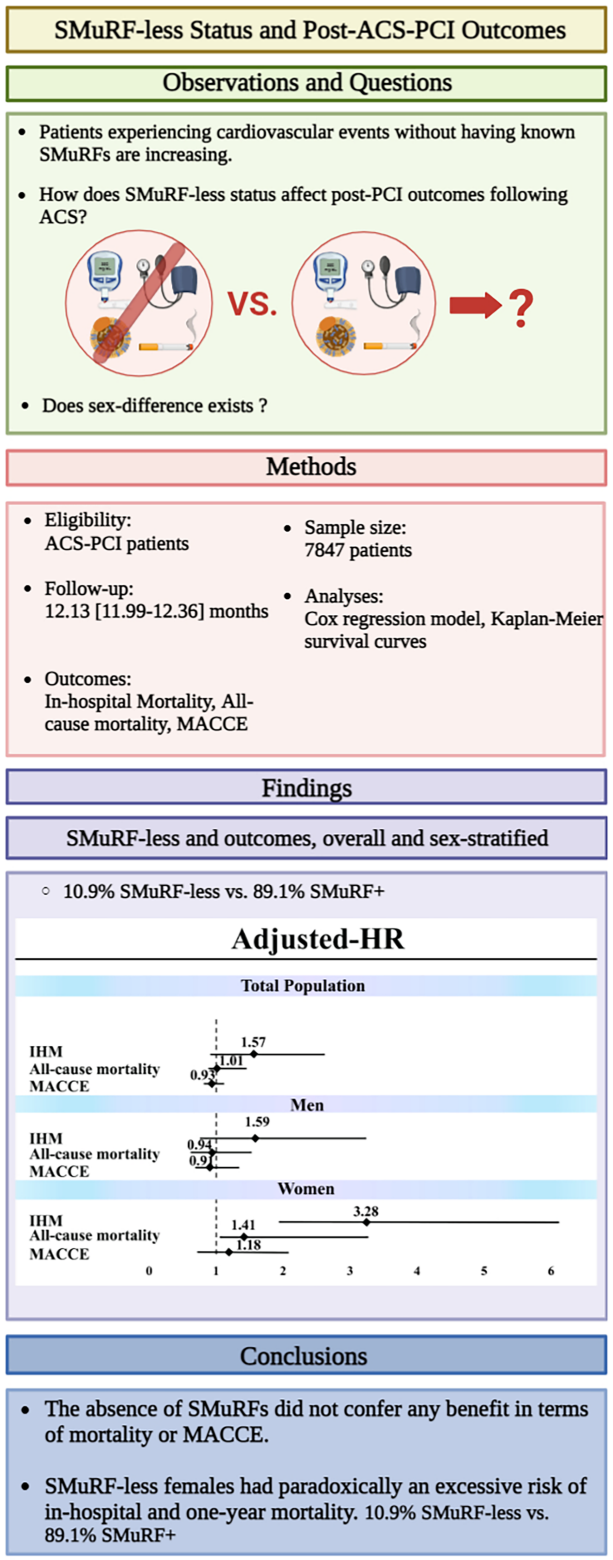
Summary of study design and findings.

In the present study, we highlighted the importance of SMuRF-less patients as an overlooked group. Figure 4 depicts a summary of study design and main findings. We found that despite traditional beliefs, post-PCI mortality and MACCE were not lower in SMuRF-less patients than in SMuRF+ patients. In SMuRF-less women, the risk of in-hospital and one-year mortality was even higher than that in SMuRF+ women. We observed a 10.9% prevalence of SMuRF-less status among patients with ACS who underwent PCI. This frequency is strongly supported by the largest original studies on this group (5) and the most recent meta-analysis on more than one million patients with ACS (21).

Despite the traditionally-held belief that a lower baseline cardiovascular risk equates to a lower risk of adverse cardiovascular outcomes, according to our results and previous studies (4–6), patients without SMuRFs did not have a reduced risk of adverse events. An enhanced risk and a not-reduced risk in SMuRF-less patients do not contradict each other since they both imply that the risk of adverse events in this group is, if not higher, as high as that in SMuRF+ patients. Various studies have indicated higher in-hospital/early mortality for SMuRF-less patients presenting with ACS, STEMI, or NSTEMI (5, 6, 22), among which is the most recent meta-analysis reporting a 57% excess risk of in-hospital mortality (21) in the ACS cohort of SMuRF-less patients. Studies with longer follow-ups are scarcer and more inconsistent, some of which have reported similar one-year mortality (23) and six-month MACCE (6), while others have indicated lower long-term (up to 12 years) mortality and MACCE among SMuRF-less patients (24). Future studies on the long-term outcomes of this group seem imperative.

Understanding the underlying pathophysiological pathways and factors contributing to the heightened susceptibility of SMuRF-less individuals to worse outcomes of coronary diseases remains an area of ongoing investigation (23). Various factors might be responsible for this notion. Our SMuRF-less individuals, consistent with previous studies (5, 23, 25), were more likely to have left anterior descending coronary artery involvement as the culprit vessel than their SMuRF+ counterparts, implying less favorable outcomes due to a larger myocardial territory at risk. Furthermore, SMuRF+ patients may benefit from the presence of coronary collaterals, particularly in cases of silent myocardial ischemia associated with established cardiovascular disease risk factors, leading to potentially lower mortality rates (26, 27). Factors such as embryologic origin (28) and biomechanical forces (29) are also potential areas of focus for better comprehending of these differences. For instance, there might exist some disparities between SMuRF-less patients and their counterpart regarding the angle between left-main and left anterior descending coronary arteries which is assumed to affect CAD progression (29). Unraveling these mechanisms will provide valuable insights into the pathogenesis of coronary atherosclerosis in SMuRF-less patients and facilitate the development of more targeted risk assessment and management strategies.

Recommended risk score models [such as the Framingham risk score and other validated algorithms centering around traditional risk factors (30, 31)] fail to truly identify these high-risk patients, thereby underestimating the risk in SMuRF-less patients. Hence, primary and secondary prevention strategies for such patients are still challenging and lacking enough evidence, necessitating new risk score models considering genetic markers and novel biomarkers. This notion is consistent with the hypothesis that new undiscovered mechanisms are involved in atherosclerosis. Genome-wide associated studies have indicated that several genetic loci linked with CHD are not related to the presence of SMuRFs and act independently in the atherosclerosis process (32). One of the other proposed reasons behind the increased/not decreased risk in this group is based on the pre-PCI Thrombolysis in myocardial infarction (TIMI) score, as SMuRF-less patients have been shown to unexpectedly have higher rates of TIMI 0/1 flow (23). Nevertheless, this was not the case with our study population.

Furthermore, SMuRF-less patients might comprise a group with missed diagnosis of traditional risk factors or those having continuous values of SMuRFs just below the usual thresholds (for example pre-diabetic patients). This group might also encompass patients with atypical or other risk factors, such as cancer, liver, autoimmune, or inflammatory diseases (22, 33). It is also worth mentioning that despite having no traditional risk factors, SMuRF-less patients had experienced the ACS episode, implying that these patients possess a highly susceptible atherosclerotic substrate independent of the central atherosclerosis risk factors. This pathway might involve autonomic dysfunction, oxidative stress, or inflammatory mechanisms (34).

Interestingly, environmental factors, such as air pollution, can play a role in this regard, as it has been shown to associate with systemic inflammation and oxidative stress (35). Therefore, the interplay between air pollution, systemic inflammation, and the susceptible atherosclerotic substrate in SMuRF-less patients may contribute to their increased risk of adverse cardiovascular events. However, further research is needed to elucidate the precise mechanisms through which air pollution influences coronary health in this population.

Notably, the association between the SMuRF-less status and considerable early mortality might be mediated through increased in-hospital complications, such as increased cardiogenic shock (21) and acute kidney injury (36). Different ACS presentations in the SMuRF-less population can also justify their not-reduced risk of adverse events as they are more likely to present with STEMI than patients with conventional risk factors (37).

Last but not least, SMuRF-less patients are less inclined to receive guideline-directed medical therapy after ACS, including statins, beta-blockers, angiotensin-converting enzyme inhibitors/ angiotensin receptor blockers, and P2Y12 inhibitors (21, 38). These medications are demonstrated to have cardioprotective effects, regardless of the presence of conventional risk factors (39, 40). Therefore, they should be equally considered in the post-ACS management in SMuRF-less patients as well.

Our variable definition, based on the Catheterization and Percutaneous Coronary Intervention (CathPCI) Registry form (16), did not allow differentiation between non-atherosclerotic conditions such as myocardial infarction with nonobstructive coronary arteries (MINOCA) or spontaneous coronary artery dissection (SCAD) and atherosclerotic ACS.

MINOCA and SCAD have a low prevalence among an older population, like ours (with a mean age of approximately 62) (41), predominantly affecting women (42–44). Furthermore, they exhibit fewer conventional cardiovascular risk factors, aligning them predominantly with the SMuRF-less women subgroup. Previous studies show comparable outcomes between SCAD-ACS and atherosclerotic-ACS patients, and a better outcome of MINOCA-ACS compared to atherosclerotic-ACS patients (45, 46). We believe that the exclusion of these patients, would not have affected our findings, due to their possibly low prevalence in our population and their similar outcomes with atherosclerotic-ACS patients.

Based on the results of the present study, SMuRF-less women had a higher risk of in-hospital and one-year all-cause mortality and similar MACCE risk than SMuRF+ women. According to our landmark analyses, their higher one-year mortality possibly originated from higher early mortality. The risk of in-hospital mortality, one-year mortality, and MACCE did not differ between SMuRF-less and SMuRF+ men.

Given the known higher risk of post-PCI adverse events and recurrent cerebrovascular events in women (47), clinicians have focused on mitigating their conventional risk factors to reduce the risk of CHD development during the past decades (48). Moreover, women usually present with atypical symptoms, which may delay appropriate invasive diagnostic and therapeutic measures (49). The diagnosis of CHD in SMuRF-less women is further delayed due to gender bias in clinical decision-making (50) and the fact that their symptoms might not be taken as seriously, especially when they do not have traditional risk factors.

Several factors, in addition to SMuRFs, are known to be associated with the increased risk of CHD and probably worse outcomes in women, including reproductive and pregnancy-related factors such as early menarche, early menopause, earlier age at first birth, hysterectomy (51), preeclampsia (52), and gestational diabetes mellitus (53). However, we had no access to these risk factors and could not assess their effect in our analyses. Further studies are warranted to evaluate how such risk factors may affect the outcomes in women with and without SMuRFs.

In conclusion, it does not seem reasonable only to consider coronary risk factors for cardiovascular outcome evaluation, especially among women. This practice can lead to the underdiagnosis and undertreatment of a considerable proportion of SMuRF-less women. Moreover, special attention should be paid to the inequities in the secondary prevention and medical management of SMuRF-less women. An equally close risk factor management and follow-ups should be enacted strictly after PCI, irrespective of sex and the SMuRF status (54).

## 5. Strengths and limitations

Although our total sample size was large, the number of SMuRF-less patients was relatively low, which might have underpowered the study to detect some statistically significant associations. Further, the follow-up period was limited; therefore, our findings cannot derive the long-term outcomes of SMuRF-less patients. Additionally, our analyses were based on the baseline presence of SMuRFs; thereby, we could not assess the impact of SMuRFs that developed during follow-ups. Albeit not evaluated in our study, the well-established importance of other modifiable risk factors, such as psychosocial factors, dietary habits, and regular physical activity should be noted when evaluating baseline risk status of the SMuRF-less population. Therefore, there is a possibility of residual risk which we could not adjust for. Furthermore, we were not able to take into account the impacts of different ACS presentation and medication use on patient outcomes. However, despite the fact that we did not include ASC treatment in our models, both SMuRF+ and SMuRF-less patients received guideline-directed medical therapy for ACS; therefore, the group are most probably comparable in this regard. We failed to collect data on several reproductive factors in women associated with cardiovascular events. Finally, we could not differentiate between patients with atherosclerotic ACS and those with less prevalent causes of ASC, such as SCAD or MINOCA. These conditions would have a low prevalence among an older population like ours, and patients with these two conditions would most probably belong to SMuRF-less women. Considering that their prognosis is comparable to (for SCAD patients) or better than (for MINOCA patients) atherosclerotic patients, excluding them from our study would probably not change the conclusions.

Despite these limitations, our study presented a novel and debatable matter, about which few studies exist. Furthermore, we comprehensively evaluated sex differences across most important outcomes in the cardiovascular literature. Finally, we performed a landmark analysis to better understand the importance of events occurring to the SMuRF-less population in the early postoperative period.

## 6. Conclusion

Almost one in 10 patients with ACS undergoing non-elective PCI had none of the traditional SMuRFs. Among this presumably lower-risk group, the absence of SMuRFs did not confer any benefit regarding in-hospital and one-year outcomes following PCI. Notably, SMuRF-less women had even paradoxically higher in-hospital and all-cause mortality. These findings indicate the following:

(A) the significance of the evidence-based treatment and secondary prevention of post-PCI ACS patients regardless of their perceived baseline lower risk and sex, (B) the need for utilizing newer markers for risk stratification among SMuRF-less patients, (C) the necessity of looking for the impact of female-specific conditions on their cardiovascular risks, and (D) the need to identify the possible disparities in medical management between men and women with ACS.

## Data Availability

The data analyzed in this study is subject to the following licenses/restrictions: Data are available upon reasonable request. Requests to access these datasets should be directed to KH, kaveh_hosseini130@yahoo.com; MJ, jameie.mana@gmail.com.

## References

[1] Grundy SM Balady GJ Criqui MH Fletcher G Greenland P Hiratzka LF Primary prevention of coronary heart disease: guidance from Framingham: a statement for healthcare professionals from the AHA task force on risk reduction. American heart association. Circulation. (1998) 97(18):1876–87. 10.1161/01.CIR.97.18.1876 9603549

[2] Ibanez B James S Agewall S Antunes MJ Bucciarelli-Ducci C Bueno H 2017 ESC guidelines for the management of acute myocardial infarction in patients presenting with ST-segment elevation: the task force for the management of acute myocardial infarction in patients presenting with ST-segment elevation of the European Society of Cardiology (ESC). Eur Heart J. (2017) 39(2):119–77. 10.1093/eurheartj/ehx393 28886621

[3] Roth GA Mensah GA Johnson CO Addolorato G Ammirati E Baddour LM Global burden of cardiovascular diseases and risk factors, 1990–2019: update from the GBD 2019 study. J Am Coll Cardiol. (2020) 76(25):2982–3021. 10.1016/j.jacc.2020.11.010 33309175 PMC7755038

[4] Vernon ST Coffey S Bhindi R Soo Hoo SY Nelson GI Ward MR Increasing proportion of ST elevation myocardial infarction patients with coronary atherosclerosis poorly explained by standard modifiable risk factors. Eur J Prev Cardiol. (2017) 24(17):1824–30. 10.1177/2047487317720287 28703626

[5] Figtree GA Vernon ST Hadziosmanovic N Sundström J Alfredsson J Arnott C Mortality in STEMI patients without standard modifiable risk factors: a sex-disaggregated analysis of SWEDEHEART registry data. Lancet. (2021) 397(10279):1085–94. 10.1016/S0140-6736(21)00272-5 33711294

[6] Vernon ST Coffey S D’Souza M Chow CK Kilian J Hyun K ST-segment-elevation myocardial infarction (STEMI) patients without standard modifiable cardiovascular risk factors-how common are they, and what are their outcomes? J Am Heart Assoc. (2019) 8(21):e013296. 10.1161/JAHA.119.013296 31672080 PMC6898813

[7] Guo Y Yin F Fan C Wang Z. Gender difference in clinical outcomes of the patients with coronary artery disease after percutaneous coronary intervention: a systematic review and meta-analysis. Medicine (Baltimore). (2018) 97(30):e11644. 10.1097/MD.0000000000011644 30045311 PMC6078653

[8] Poorhosseini H Abbasi SH. The Tehran heart center. Eur Heart J. (2018) 39(29):2695–6. 10.1093/eurheartj/ehy369 30289514

[9] Expert Panel on Detection, Evaluation, and Treatment of High Blood Cholesterol in Adults. Executive summary of the third report of the national cholesterol education program (NCEP) expert panel on detection, evaluation, and treatment of high blood cholesterol in adults (adult treatment panel III). JAMA. (2001) 285(19):2486–97. 10.1001/jama.285.19.2486 11368702

[10] Grundy SM Cleeman JI Merz CN Brewer HB Jr Clark LT Hunninghake DB Implications of recent clinical trials for the national cholesterol education program adult treatment panel III guidelines. Circulation. (2004) 110(2):227–39. 10.1161/01.CIR.0000133317.49796.0E 15249516

[11] Weber MA Schiffrin EL White WB Mann S Lindholm LH Kenerson JG Clinical practice guidelines for the management of hypertension in the community: a statement by the American society of hypertension and the international society of hypertension. J Clin Hyperten (Greenwich, Conn). (2014) 16(1):14–26. 10.1111/jch.12237 PMC8031779 24341872

[12] American Diabetes Association. Diagnosis and classification of diabetes Mellitus. Diabetes Care. (2009) 32(Suppl 1):S62–S7. 10.2337/dc09-S062 19118289 PMC2613584

[13] Wright RS Anderson JL Adams CD Bridges CR Casey DE Ettinger SM 2011 ACCF/AHA focused update of the guidelines for the management of patients with unstable angina/ non–ST-elevation myocardial infarction (updating the 2007 guideline). Circulation. (2011) 123(18):2022–60. 10.1161/CIR.0b013e31820f2f3e 21444889

[14] O’Gara PT Kushner FG Ascheim DD Casey DE Chung MK Lemos J 2013 ACCF/AHA guideline for the management of ST-elevation myocardial infarction. Circulation. (2013) 127(4):e362–425. 10.1161/CIR.0b013e3182742cf6 23247304

[15] Levine GN Bates ER Blankenship JC Bailey SR Bittl JA Cercek B 2011 ACCF/AHA/SCAI guideline for percutaneous coronary intervention: executive summary: a report of the American College of Cardiology Foundation/American Heart Association Task Force on practice guidelines and the society for cardiovascular angiography and interventions. Circulation. (2011) 124(23):2574–609. 10.1161/CIR.0b013e31823a5596 22064598

[16] American College of Cardiology Foundation. NCDR CathPCI Registry. rptDataCollectorDictionary_nocodes (ncdr.com) (2011). Available at: https://www.ncdr.com/WebNCDR/docs/default-source/public-data-collection-documents/cathpci_v4_codersdictionary_4-4.pdf?sfvrsn=b84d368e_2

[17] Bagheri J Jameie M Saryazdi ZD Jalali A Rezaee M Pashang M Coronary artery bypass graft surgery after primary percutaneous coronary intervention in patients with ST-elevation myocardial infarction. Heart Lung Circ. (2023) 32(10):1257–68. 10.1016/j.hlc.2023.08.005 37741752

[18] TT. A Package for Survival Analysis in R_. R package version 3.2-10. 2021. (2021).

[19] Alboukadel Kassambara MKaPB. survminer: Drawing Survival Curves using ‘ggplot2’. R package version 0.4.9. (2021).

[20] Wickham H. Ggplot2: Elegant graphics for data analysis. New York: Springer-Verlag (2016).

[21] Kong G Chin YH Chong B Goh RSJ Lim OZH Ng CH Higher mortality in acute coronary syndrome patients without standard modifiable risk factors: results from a global meta-analysis of 1,285,722 patients. Int J Cardiol. (2023) 371:432–40. 10.1016/j.ijcard.2022.09.062 36179904

[22] Suzuki S Saito Y Yamashita D Matsumoto T Sato T Wakabayashi S Clinical characteristics and prognosis of patients with No standard modifiable risk factors in acute myocardial infarction. Heart Lung Circ. (2022) 31(9):1228–33. 10.1016/j.hlc.2022.06.666 35843858

[23] Figtree GA Redfors B Kozor R Vernon ST Grieve SM Mazhar J Clinical outcomes in patients with ST-segment elevation MI and no standard modifiable cardiovascular risk factors. JACC Cardiovasc Interv. (2022) 15(11):1167–75. 10.1016/j.jcin.2022.03.036 35680197

[24] Figtree GA Vernon ST Hadziosmanovic N Sundström J Alfredsson J Nicholls SJ Mortality and cardiovascular outcomes in patients presenting with non-ST elevation myocardial infarction despite No standard modifiable risk factors: results from the SWEDEHEART registry. J Am Heart Assoc. (2022) 11(15):e024818. 10.1161/JAHA.121.024818 35876409 PMC9375489

[25] Mazhar J Ekström K Kozor R Grieve SM Nepper-Christensen L Ahtarovski KA Cardiovascular magnetic resonance characteristics and clinical outcomes of patients with ST-elevation myocardial infarction and no standard modifiable risk factors-A DANAMI-3 substudy. Front Cardiovasc Med. (2022) 9:945815. 10.3389/fcvm.2022.945815 35990971 PMC9383416

[26] Pei J Wang X Xing Z. Traditional cardiovascular risk factors and coronary collateral circulation: a meta-analysis. Front Cardiovasc Med. (2021) 8:743234. 10.3389/fcvm.2021.743234 34805302 PMC8595282

[27] Sia C-H Ko J Zheng H Ho AF-W Foo D Foo L-L Comparison of mortality outcomes in acute myocardial infarction patients with or without standard modifiable cardiovascular risk factors. Front Cardiovasc Med. (2022) 9:876465. 10.3389/fcvm.2022.876465 35497977 PMC9047915

[28] Ishii Y Langberg J Rosborough K Mikawa T. Endothelial cell lineages of the heart. Cell Tissue Res. (2009) 335(1):67–73. 10.1007/s00441-008-0663-z 18682987 PMC2729171

[29] Moon SH Byun JH Kim JW Kim SH Kim KN Jung JJ Clinical usefulness of the angle between left main coronary artery and left anterior descending coronary artery for the evaluation of obstructive coronary artery disease. PLoS One. (2018) 13(9):e0202249-e. 10.1371/journal.pone.0202249 30212455 PMC6136703

[30] Ridker PM Buring JE Rifai N Cook NR. Development and validation of improved algorithms for the assessment of global cardiovascular risk in women: the reynolds risk score. JAMA. (2007) 297(6):611–9. 10.1001/jama.297.6.611 17299196

[31] Wilson PW D'Agostino RB Levy D Belanger AM Silbershatz H Kannel WB. Prediction of coronary heart disease using risk factor categories. Circulation. (1998) 97(18):1837–47. 10.1161/01.CIR.97.18.1837 9603539

[32] Deloukas P Kanoni S Willenborg C Farrall M Assimes TL Thompson JR Large-scale association analysis identifies new risk loci for coronary artery disease. Nat Genet. (2013) 45(1):25–33. 10.1038/ng.2480 23202125 PMC3679547

[33] Sokhal BS Matetić A Paul TK Velagapudi P Lambrinou E Figtree GA Management and outcomes of patients admitted with type 2 myocardial infarction with and without standard modifiable risk factors. Int J Cardiol. (2023) 371:391–6. 10.1016/j.ijcard.2022.09.037 36130622

[34] Arnold N Lechner K Waldeyer C Shapiro MD Koenig W. Inflammation and cardiovascular disease: the future. European Cardiology. (2021) 16:e20. 10.15420/ecr.2020.50 34093741 PMC8157394

[35] Montone RA Camilli M Russo M Termite C La Vecchia G Iannaccone G Air pollution and coronary plaque vulnerability and instability: an optical coherence tomography study. JACC Cardiovasc Imaging. (2022) 15(2):325–42. 10.1016/j.jcmg.2021.09.008 34656488

[36] Shamaki GR Safiriyu I Kesiena O Mbachi C Anyanwu M Zahid S Prevalence and outcomes in STEMI patients without standard modifiable cardiovascular risk factors: a national inpatient sample analysis. Curr Probl Cardiol. (2022) 47(11):101343. 10.1016/j.cpcardiol.2022.101343 35934021

[37] Sheikh S Peerwani G Hanif B Virani S. Clinical characteristics, management, and 5-year survival compared between no standard modifiable risk factor (SMuRFless) and ≥1 SMuRF ACS cases: an analysis of 15,051 cases from Pakistan. BMC Cardiovasc Disord. (2023) 23(1):320. 10.1186/s12872-023-03355-z 37355597 PMC10290799

[38] Yusuf S Joseph P Rangarajan S Islam S Mente A Hystad P Modifiable risk factors, cardiovascular disease, and mortality in 155 722 individuals from 21 high-income, middle-income, and low-income countries (PURE): a prospective cohort study. Lancet. (2020) 395(10226):795–808. 10.1016/S0140-6736(19)32008-2 31492503 PMC8006904

[39] Amsterdam EA Wenger NK Brindis RG Casey DE Jr Ganiats TG Holmes DR Jr 2014 AHA/ACC guideline for the management of patients with non-ST-elevation acute coronary syndromes: a report of the American College of Cardiology/American Heart Association Task Force on practice guidelines. J Am Coll Cardiol. (2014) 64(24):e139–228. 10.1016/j.jacc.2014.09.017 25260718

[40] Collet J-P Thiele H Barbato E Barthélémy O Bauersachs J Bhatt DL 2020 ESC guidelines for the management of acute coronary syndromes in patients presenting without persistent ST-segment elevation. Eur Heart J. (2021) 42(14):1289–367. 10.1093/eurheartj/ehaa575 32860058

[41] Niccoli G Camici PG. Myocardial infarction with non-obstructive coronary arteries: what is the prognosis? Eur Heart J Suppl. (2020) 22(Suppl E):E40–e5. 10.1093/eurheartj/suaa057 32523437 PMC7270909

[42] Giacoppo D Capodanno D Dangas G Tamburino C. Spontaneous coronary artery dissection. Int J Cardiol. (2014) 175(1):8–20. 10.1016/j.ijcard.2014.04.178 24861255

[43] Almasi A Mansouri P Jameie M Yadangi S Parapary SH Mohsenizadeh SA Clinical features and prognoses of middle-aged women with ST-elevation myocardial infarction with a focus on spontaneous coronary artery dissection. Crit Pathw Cardiol. (2022) 21(1):18–23. 10.1097/HPC.0000000000000275 34919066

[44] Pasupathy S Air T Dreyer RP Tavella R Beltrame JF. Systematic review of patients presenting with suspected myocardial infarction and nonobstructive coronary arteries. Circulation. (2015) 131(10):861–70. 10.1161/CIRCULATIONAHA.114.011201 25587100

[45] Adams H Paratz E Somaratne J Layland J Burns A Palmer S Different patients, different outcomes: a case-control study of spontaneous coronary artery dissection versus acute coronary syndrome. J Interv Cardiol. (2018) 31(1):41–7. 10.1111/joic.12447 28940292

[46] Daoulah A Al-Faifi SM Madan M Arafat AA Hersi AS Alasmari A Clinical presentation and outcome of patients with spontaneous coronary artery dissection versus atherosclerotic coronary plaque dissection. Crit Pathw Cardiol. (2021) 20(1):36–43. 10.1097/HPC.0000000000000233 32657974

[47] Mehilli J Presbitero P. Coronary artery disease and acute coronary syndrome in women. Heart. (2020) 106(7):487–92. 10.1136/heartjnl-2019-315555 31932287

[48] Khandelwal A Bakir M Bezaire M Costello B Gomez JMD Hoover V Managing ischemic heart disease in women: role of a women’s heart center. Curr Atheroscler Rep. (2021) 23(10):56. 10.1007/s11883-021-00956-x 34345945 PMC8331213

[49] Rao U Buchanan GL Hoye A. Outcomes after percutaneous coronary intervention in women: are there differences when compared with men? Interv Cardiol. (2019) 14(2):70–5. 10.15420/icr.2019.09 31178932 PMC6545995

[50] Maserejian NN Link CL Lutfey KL Marceau LD McKinlay JB. Disparities in physicians’ interpretations of heart disease symptoms by patient gender: results of a video vignette factorial experiment. J Women’s Health. (2009) 18(10):1661–7. 10.1089/jwh.2008.1007 PMC2825679 19785567

[51] Peters SA Woodward M. Women’s reproductive factors and incident cardiovascular disease in the UK biobank. Heart. (2018) 104(13):1069–75. 10.1136/heartjnl-2017-312289 29335253

[52] McDonald SD Malinowski A Zhou Q Yusuf S Devereaux PJ. Cardiovascular sequelae of preeclampsia/eclampsia: a systematic review and meta-analyses. Am Heart J. (2008) 156(5):918–30. 10.1016/j.ahj.2008.06.042 19061708

[53] Tobias DK Stuart JJ Li S Chavarro J Rimm EB Rich-Edwards J Association of history of gestational diabetes with long-term cardiovascular disease risk in a large prospective cohort of US women. JAMA Intern Med. (2017) 177(12):1735–42. 10.1001/jamainternmed.2017.2790 29049820 PMC5820722

[54] Lee MT Mahtta D Ramsey DJ Liu J Misra A Nasir K Sex-related disparities in cardiovascular health care among patients with premature atherosclerotic cardiovascular disease. JAMA Cardiol. (2021) 6(7):782–90. 10.1001/jamacardio.2021.0683 33881448 PMC8060887

